# fNIRS-based brain-computer interfaces: a review

**DOI:** 10.3389/fnhum.2015.00003

**Published:** 2015-01-28

**Authors:** Noman Naseer, Keum-Shik Hong

**Affiliations:** ^1^Department of Cogno-Mechatronics Engineering, Pusan National UniversityBusan, Republic of Korea; ^2^School of Mechanical Engineering, Pusan National UniversityBusan, Republic of Korea

**Keywords:** brain-computer interface, functional near-infrared spectroscopy (fNIRS), feature extraction, feature classification, physiological noise, brain-machine interface

## Abstract

A brain-computer interface (BCI) is a communication system that allows the use of brain activity to control computers or other external devices. It can, by bypassing the peripheral nervous system, provide a means of communication for people suffering from severe motor disabilities or in a persistent vegetative state. In this paper, brain-signal generation tasks, noise removal methods, feature extraction/selection schemes, and classification techniques for fNIRS-based BCI are reviewed. The most common brain areas for fNIRS BCI are the primary motor cortex and the prefrontal cortex. In relation to the motor cortex, motor imagery tasks were preferred to motor execution tasks since possible proprioceptive feedback could be avoided. In relation to the prefrontal cortex, fNIRS showed a significant advantage due to no hair in detecting the cognitive tasks like mental arithmetic, music imagery, emotion induction, etc. In removing physiological noise in fNIRS data, band-pass filtering was mostly used. However, more advanced techniques like adaptive filtering, independent component analysis (ICA), multi optodes arrangement, etc. are being pursued to overcome the problem that a band-pass filter cannot be used when both brain and physiological signals occur within a close band. In extracting features related to the desired brain signal, the mean, variance, peak value, slope, skewness, and kurtosis of the noised-removed hemodynamic response were used. For classification, the linear discriminant analysis method provided simple but good performance among others: support vector machine (SVM), hidden Markov model (HMM), artificial neural network, etc. fNIRS will be more widely used to monitor the occurrence of neuro-plasticity after neuro-rehabilitation and neuro-stimulation. Technical breakthroughs in the future are expected via bundled-type probes, hybrid EEG-fNIRS BCI, and through the detection of initial dips.

## Introduction

A brain-computer interface (BCI) system provides its users with control channels that are independent of the brain's output channels (i.e., the peripheral nervous system and muscles) (Wolpaw et al., 2002). Such systems can be used as a means for communications and restoration of motor functions (through a neuroprosthesis) for people with motor disorders such as amyotrophic lateral sclerosis (ALS) and spinal cord injury, and/or people in the persistent locked-in state (LIS). It can also be used as a neurorehabilitation tool to improve motor and/or cognitive performance of such people.

**Figure 1:**
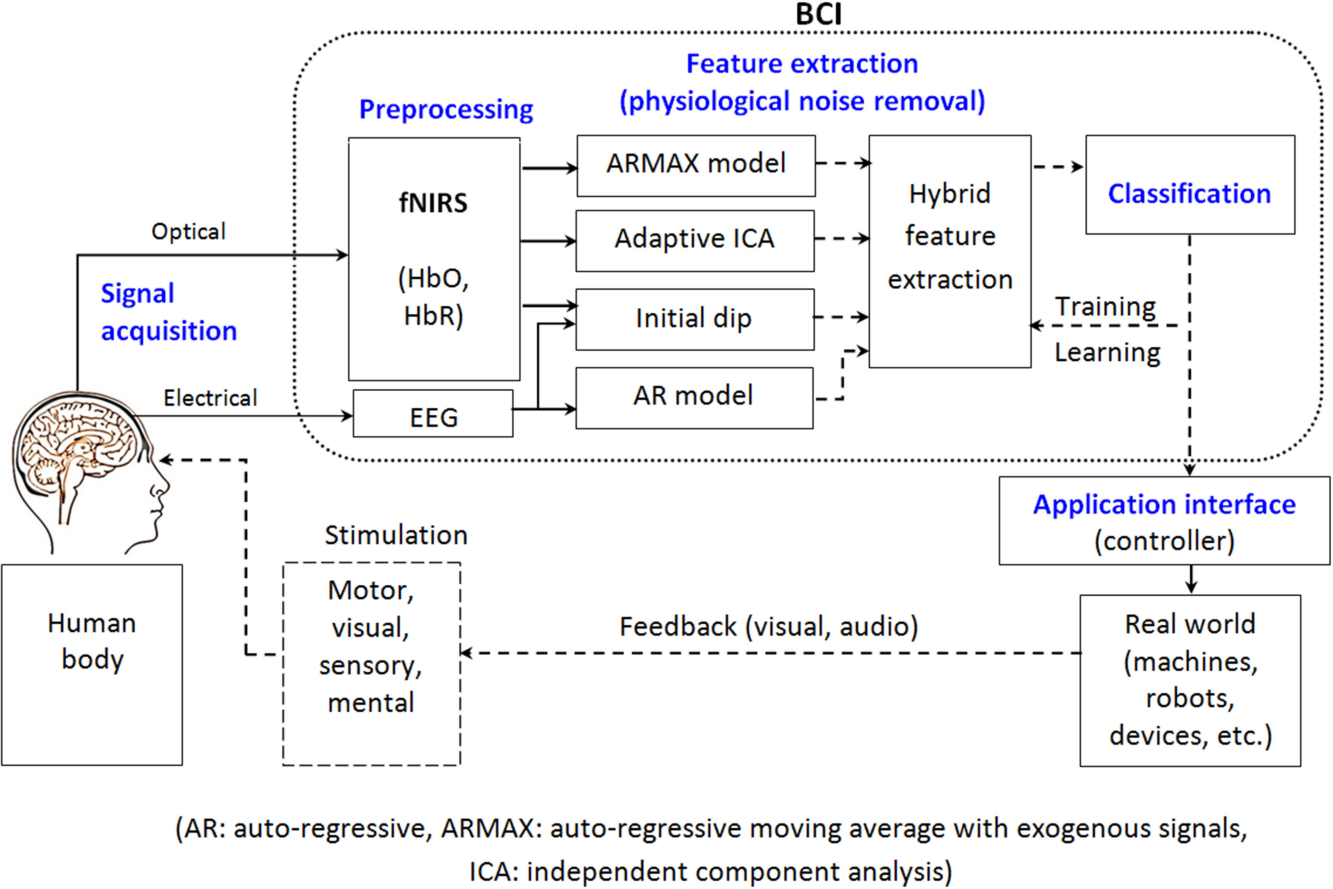
**Schematic of a hybrid fNIRS-EEG BCI**.

A typical BCI system consists of five stages (see Figure 1): brain-signal acquisition, preprocessing, feature extraction/selection, classification, and application interface. In the first brain-signal acquisition stage, suitable signals are acquired using an appropriate brain-imaging modality. Since the acquired signals are normally weak and contain noises (physiological and instrumental) and artifacts, preprocessing is needed, which is the second stage. In the third stage, some useful data so called “features” are extracted. These features, in the fourth stage, are classified using a suitable classifier. Finally, in the fifth stage, the classified signals are transmitted to a computer or other external devices for generating the desired control commands to the devices. In neurofeedback applications, a real-time display of brain activity is desirable, which enables self-regulation of brain functions. Figure 1 depicts a schematic of (hybrid) functional near-infrared spectroscopy (fNIRS) and electroencephalography (EEG) BCI.

Several modalities have been used for brain signal acquisition, which include EEG (Wolpaw et al., 2002; Turnip et al., 2011; Turnip and Hong, 2012; Wang et al., 2012; Hwang et al., 2013; Kleih and Kubler, 2013; Ko and Sim, 2013; Hammer et al., 2014; Kim et al., 2014; Soekadar et al., 2014), magnetoencephalography (MEG) (Mellinger et al., 2007; Buch et al., 2008; Sardouie and Shamsollahi, 2012), functional magnetic resonance imaging (fMRI) (Weiskopf et al., 2004; LaConte, 2011; van der Heiden et al., 2014), and fNIRS (Ferrari et al., 1985, 2004; Kato et al., 1993; Hu et al., 2013; Bhutta et al., 2014; Rea et al., 2014; Santosa et al., 2014). Among them, fNIRS is relatively new, which uses near-infrared-range light (usually of 650~1000 nm wavelength) to measure the concentration changes of oxygenated hemoglobin (HbO) and deoxygenated hemoglobin (HbR) (Villringer et al., 1993; Hoshi et al., 1994; Hoshi and Tamura, 1997; Villringer and Chance, 1997; Boas et al., 2004a,b; Hong and Nguyen, 2014). Its main advantages are relatively low cost, portability, safety, low noise (compared to fMRI), and easiness to use. Unlike EEG and MEG, its data are not much susceptible to electrical noise, since it is an optical imaging modality. fNIRS measures the blood flow changes in the local capillary network caused by neuron firings. Since the hemoglobin is an oxygen carrier, the changes of HbO and HbR concentration levels after a neuronal activation can be related to the relevant neuronal firings. fNIRS uses near-infrared (NI) light emitter-detector pairs operating with two or more wavelengths. The NI light emitted into the scalp diffuses through the brain tissues resulting in multiple scattering of photons. Some of these photons exit the head after passing through the cortical region of the brain, wherein the chromophores (i.e., HbO and HbR) are changing in time. These exited photons are then detected by using strategically positioned detectors. Since HbO and HbR have different absorption coefficients for different wavelengths of NI light, the relationship between the exiting-photon intensity and the incident-photon intensity can be used to calculate the changes of the concentrations of HbO and HbR [Δ*c*_HbO_(*t*) and Δ*c*_HbR_(*t*)] along the path of the photons by applying the modified Beer-Lamberts law (Delpy et al., 1988).

The principle of fNIRS measurement, first reported by Jobsis (1977), has been applied to the study of cerebral hemodynamics for more than two decades, even though its BCI use is only a few years old. The first study who demonstrated the feasibility of fNIRS for BCI was Coyle et al. (2004). They asked the subjects to perform motor imagery of continuous squeezing and releasing of a soft ball. Based on the activity threshold of Δ*c*_HbO_(*t*), they determined whether the brain was activated or at rest.

In 2007, three studies demonstrated the feasibility of controlling the output of fNIRS BCI: Coyle et al. (2007) used a custom-built fNIRS system (named Mindswitch) to test on-off control. Their protocol consisted of two options alternately presented to the subjects: When a desired option was highlighted, the subject performed motor imagery of squeezing and releasing a soft ball to enhance the HbO signals in the motor cortex and, in this way, expressed their choice mentally. The signals during motor imagery were classified against those during the rest period with an average accuracy of more than 80%. Sitaram et al. (2007) showed that fNIRS signal patterns during execution movement and imagery were distinguishable with the accuracy of 80% (or above) using support vector machines (SVM) and hidden Markov model (HMM). On the other hand, the first investigation on ALS patients was done by Naito et al. (2007): Forty ALS patients (17 of them were totally locked-in) were asked to encode their response to several questions as “yes” or “no.” They were requested to respond “yes” by performing mental calculation, music imagery and other such tasks, and to respond “no” by remaining relaxed. The instantaneous amplitude and phase of the light-intensity signals were then used as the features for a quadratic discriminant analysis classifier, which successfully decoded the responses of 70% of the ALS patients who were not totally locked-in. However, for totally locked-in ALS patients, the method worked only 40% of subjects (with the classification accuracy of about 80%).

In 2008, Utsugi et al. (2008) showed the feasibly of a “Go-Stop” control. They measured the spatiotemporal averages of Δ*c*_HbO_(*t*) and Δ*c*_HbR_(*t*) arising from mental calculations. Bauernfeind et al. (2008) developed an fNIRS system and reported that changes in Δ*c*_HbO_(*t*) and Δ*c*_HbR_(*t*) were observed during mental arithmetic tasks over the prefrontal cortex. The measured signals were relatively stable across 13 subjects. Based on that, the authors suggested its application to BCI.

In 2009, Luu and Chau (2009) demonstrated the preference-decoding possibilities using fNIRS signals acquired from the prefrontal cortex. Nine subjects were asked to mentally evaluate two presented drinks and decide which one they preferred. Instead of using a specific activity to choose the preferred drink, they used the direct neural correlates in decision making. The accuracy of this preference decoding, using light-intensity signals directly and linear discriminant analysis (LDA), was around 80%. In the same year, Tai and Chau (2009) showed the feasibility for BCI development of fNIRS-signal classification from emotion-induction tasks. The subjects preformed several trials of positive- and negative-emotion-induction tasks, and the optimal features were selected using a genetic algorithm. Then, LDA and SVM were used to classify different sets of features to the average accuracies ranging from 75 to 94%. Since 2009, several studies have successfully demonstrated the use of fNIRS for efficient BCI. Although EEG-based BCIs are most common non-invasive versions, the trend of using fNIRS for BCI is continuously increasing.

## Brain-signal acquisition

**Figure 2:**
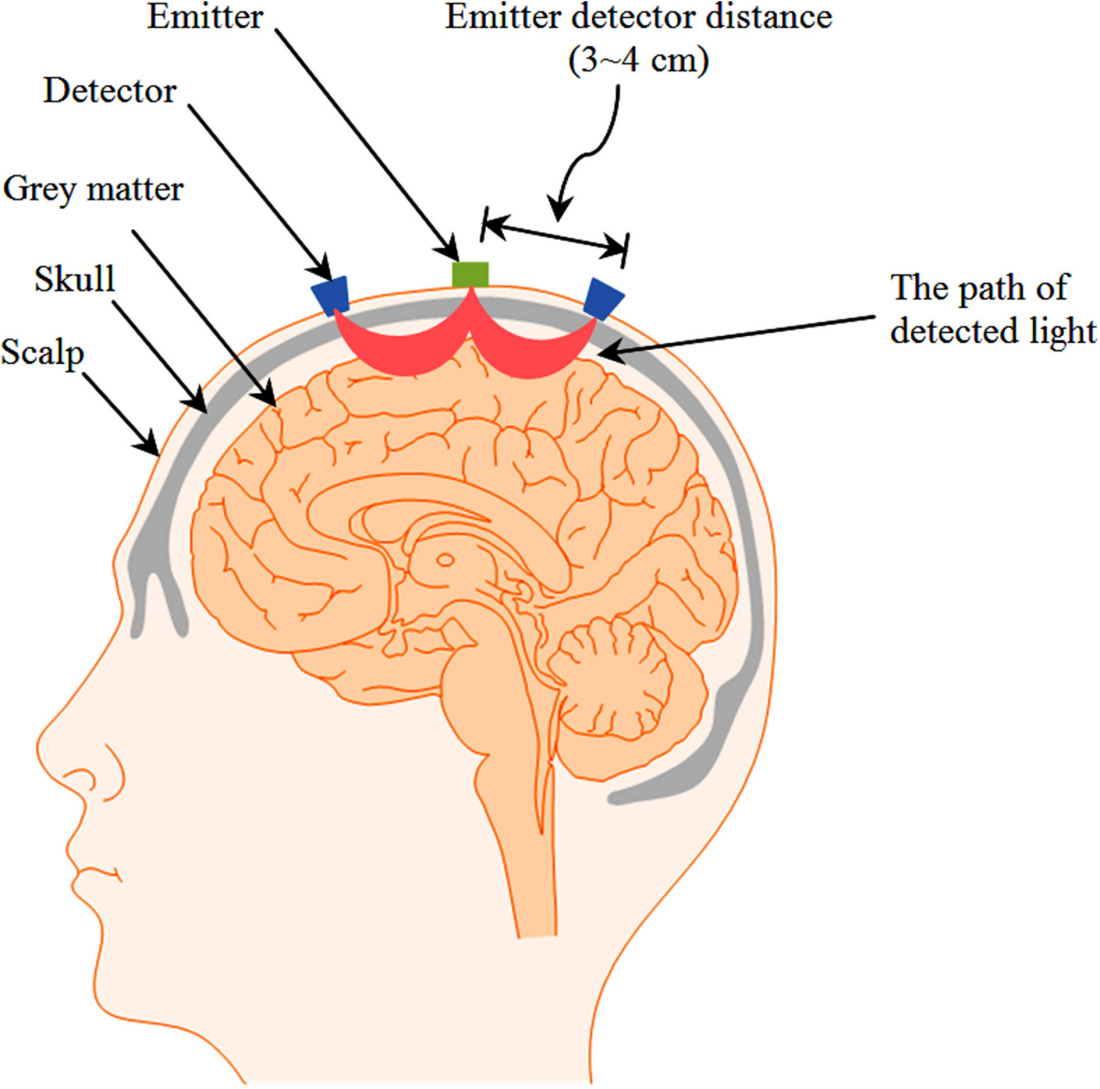
**Example of emitter-detector pairs showing the banana-shaped paths of light**.

BCI uses brain signals to collect information on the user's intensions. The first step in developing an fNIRS-BCI system is to acquire suitable brain signals. The two most common brain areas are the primary motor cortex and the prefrontal cortex. Signals corresponding to motor execution and motor imagery tasks are acquired from the motor cortex; whereas those corresponding to mental arithmetic, mental counting, music imagery, landscape imagery, etc. are acquired from the prefrontal cortex. Although several different emitter-detector configurations have been used in these two areas, the emitter-detector distance is usually kept within a specific range, as it plays an important role in fNIRS measurement. For example, an increase in emitter-detector distance corresponds to an increase in imaging depth (McCormick et al., 1992). To measure hemodynamic response signals from the cortical areas, an emitter-detector separation of around 3 cm was suggested (Gagnon et al., 2012). A separation of less than 1 cm might contain only skin-layer contribution, whereas that of more than 5 cm might result in weak and therefore unusable signals (Gratton et al., 2006). A typical emitter-detector configuration on the head and the paths traveled by light to reach two detectors are shown in Figure 2. A suitable number of emitter/detector pairs for adequate extraction of neuronal activity vary depending on the type of brain signals that are used for BCI purpose. For the prefrontal cortex, 3 emitters and 8 detectors may be enough to adequately acquire most brain signals corresponding to prefrontal tasks (Luu and Chau, 2009; Power et al., 2010, 2011, 2012a,b; Khan et al., 2014; Naseer et al., 2014). For brain activities corresponding to motor cortex tasks, 6 emitters and 6 detectors can cover the entire motor cortex. In the previous studies, 4 emitters and 4 detectors (Sitaram et al., 2007), 6 emitters and 6 detectors (Naseer and Hong, 2013), and 5 emitters and 4 detectors have been applied to acquire motor-cortex activities.

### Motor cortex activities

Activities from the primary motor cortex are a good choice for fNIRS-BCI application, as they are natural means of providing BCI control over external devices. Moreover, these might also be useful from the perspective of neurorehabilitation. The two most commonly acquired activities from the motor cortex are motor execution and motor imagery.

#### Motor execution

The motor execution task stands for moving a body part to activate the motor cortex, which involves the development of muscular tensions through muscular actions. Since motor execution involves contraction of muscles, motor execution-based BCIs are affected by proprioceptive feedback from contracting muscles and, therefore, the neuronal modulation may not be solely from the central nervous system. Several motor execution tasks including finger tapping (Cui et al., 2010a,b; Seo et al., 2012), hand tapping (Hai et al., 2013; Khan et al., 2014), arm lifting (Shin and Jeong, 2014), knee extension (Shin and Jeong, 2014) and hand grasping/gripping (Nagaoka et al., 2010; Fazli et al., 2012) have been used in the previous studies.

#### Motor imagery

Motor imagery can be defined as a covert cognitive process of kinesthetic imagining of the movement of one's own body part without the involvement of muscular tension, contraction or flexion. Since the primary objective of BCI is to form a communication pathway for motor-disabled people, motor imagery is one of the most commonly utilized tasks in fNIRS-BCI. The motor imagery tasks include imagination of the squeezing of a soft ball (Coyle et al., 2004, 2007; Stangl et al., 2013), covert imagery of a simple or complex sequence of finger tapping (Sitaram et al., 2007; Holper and Wolf, 2011), imagination of feet tapping (Kaiser et al., 2014), imagination of hand grasping/gripping (Nagaoka et al., 2010; Fazli et al., 2012; Kaiser et al., 2014), imagination of wrist flexion (Naseer and Hong, 2013), imagination of flexion and extension of elbow (Mihara et al., 2013), and folding and unfolding of specific fingers (Mihara et al., 2013). Unlike motor execution tasks, the motor imagery signals are free of proprioceptive feedback.

### Prefrontal cortex activities

The activities in the prefrontal cortex are also a good choice for fNIRS-BCI, because they involve less motion artifacts and signal attenuation due to the slippage in hairs. Also, they are likely to be more effective in the case of motor-function related disability. Given these advantages, most studies have used the prefrontal activities showing promising results (Naito et al., 2007; Bauernfeind et al., 2008, 2011; Utsugi et al., 2008; Luu and Chau, 2009; Power et al., 2010, 2011, 2012a,b; Abibullaev et al., 2011; Falk et al., 2011; Tanaka and Katura, 2011; Abibullaev and An, 2012; Adhika et al., 2012; Chan et al., 2012; Hu et al., 2012; Moghimi et al., 2012; Sagara and Kido, 2012; Faress and Chau, 2013; Power and Chau, 2013; Stangl et al., 2013; Hwang et al., 2014; Naseer et al., 2014; Schudlo and Chau, 2014; Hong et al., 2015). Some of the commonly used prefrontal activities for fNIRS-BCI are mental arithmetic, music imagery, mental counting, and landscape imagery.

#### Mental arithmetic

Mental arithmetic (sometimes called mental calculation) means performing covert calculation using the brain without any help in the form of paper, pen, calculator, computer, etc. It activates the prefrontal cortex. Since it does not involve any body movement, it is widely used for fNIRS-BCI. A number of studies have successfully demonstrated its feasibility as a mental task for BCI (Naito et al., 2007; Bauernfeind et al., 2008, 2011; Utsugi et al., 2008; Power et al., 2010, 2011, 2012a,b; Adhika et al., 2012; Sagara and Kido, 2012; Power and Chau, 2013; Stangl et al., 2013; Hwang et al., 2014; Naseer et al., 2014; Hong et al., 2015). Mental arithmetic entails mental multiplication (Hwang et al., 2014) or other arithmetic tasks. However, the most commonly utilized mental arithmetic is backwards subtraction, which involves subtraction of a small number (for example, a two-digit number) from a large number (for example, a three-digit number) with successive subtraction of a randomly appearing small number from the result of the previous subtraction (e.g., 450-15, 435-10, 425-19, etc.) (Power et al., 2010; Hwang et al., 2014; Naseer et al., 2014).

#### Music imagery

Music imagery (also called mental singing) consists of organizing and analyzing music in the brain without any external auditory stimulus. Naito et al. (2007), Power et al. (2010), Falk et al. (2011), Power et al. (2011), Chan et al. (2012) and Hwang et al. (2014) successfully demonstrated music imagery as a brain activity that can be effectively used for fNIRS-BCI.

#### Other prefrontal activities

Besides mental arithmetic and music imagery, various other tasks in the prefrontal cortex have been shown to work well. These include mental counting (Naito et al., 2007; Khan et al., 2014), landscape imagery (Naito et al., 2007), mental character writing (Hwang et al., 2014), object rotation (Abibullaev et al., 2011; Abibullaev and An, 2012; Faress and Chau, 2013; Hwang et al., 2014), change-detection tasks (Tanaka and Katura, 2011), labyrinth tasks (Misawa et al., 2012), and emotion-induction tasks (Tai and Chau, 2009; Moghimi et al., 2012). Some studies have demonstrated direct decoding of neural correlates corresponding to subjective preferences (Luu and Chau, 2009), deception (Hu et al., 2012), visual stimuli (Faress and Chau, 2013), and others (Ayaz et al., 2009, 2012).

The best selection of optimal mental activities for the improvement of classification accuracy remains an open question. Hwang et al. (2014) evaluated the use of a variety of mental task combinations for BCI. These tasks included motor imagery (right- and left-hand imagery and foot imagery), mental singing, mental arithmetic (multiplication and subtraction), mental rotation, and mental character writing. Out of the 28 different combinations tested, the mental arithmetic/mental rotation and mental arithmetic/right-hand motor imagery combinations yielded the best LDA classification results using mean hemoglobin concentration values. Prefrontal activities have been used in more than half of fNIRS-BCI studies, owing primarily to the easy application of fNIRS to the prefrontal area. Activity selection, however, depends on the given fNIRS-BCI application. For example, for the purposes of limb neurorehabilitation, it is desirable to use motor cortex activities.

## Preprocessing

The acquired fNIRS signals can contain various noises, which can be categorized into instrumental noise, experimental error, and physiological noise. Since the instrumental noise and experimental error are not related to the brain activities, it is better to remove them prior to converting the raw optical density signals to the concentration changes of HbO and HbR through the modified Beer-Lambert law (Huppert et al., 2009).

### Removal of instrumental noise

Instrumental noise is the noise of fNIRS signals present in hardware or caused by the surrounding environment (i.e., instrumental degradation is an example). It usually involves (constant) high frequencies. Such high frequency can be easily removed by a low-pass filter (for instance, 3~5 Hz of cutoff frequency). Furthermore, by minimizing the variation of the external light, instrument noise can be significantly reduced.

### Removal of experimental errors

Experimental errors include motion artifacts like head motions, which causes the movement of optodes from the assigned positions. This can cause a sudden change in the light intensity resulting in a spike-like noise. Several methods for motion-artifact correction have been proposed in the literature; the Wiener filtering-based method (Izzetoglu et al., 2005), eigenvector-based spatial filtering (i.e., principle component analysis (PCA)-based filtering) (Zhang et al., 2005), wavelet-analysis-based methods (Sato et al., 2006; Power et al., 2010), Savitzky-Golay type filters (Hai et al., 2013; Shin and Jeong, 2014), and others (Cui et al., 2010a,b; Fekete et al., 2011; Cooper et al., 2012). Please see Cooper et al. (2012) for thorough comparison of various techniques.

## Physiological noise

Physiological noises include those due to heartbeat (1~1.5 Hz), respiration (0.2~0.5 Hz), Mayer waves (~0.1 Hz), which are related to blood pressure fluctuations (Boas et al., 2004a,b; Zhang et al., 2005; Franceschini et al., 2006; Huppert et al., 2009). Several methods including band-pass filtering, adaptive filtering, PCA, and independent component analysis (ICA) have been used to remove them.

### Band-pass filtering

Since the frequency ranges of aforementioned physiological signals are usually known, a band-pass filter can be an effective means. Some fNIRS-BCI studies have shown promising results using a simple low-pass, or a high-pass, or a band-pass filtering to remove physiological noises (Coyle et al., 2004, 2007; Naito et al., 2007; Sitaram et al., 2007; Bauernfeind et al., 2008; Luu and Chau, 2009; Power et al., 2010, 2011; Hu et al., 2012; Liu et al., 2013; Hong et al., 2015).

Various cut-off frequencies for band-pass filtering have been reported in the literature: For example, Luu and Chau (2009), Power et al. (2011), Hu et al. (2012) and Tomita et al. (2014) have used the frequency bands of 0.01~0.8 Hz, 0.1~0.5 Hz, 0.01~0.2 Hz, and 0.1~0.5 Hz, respectively. In general, the band of 0.1~0.4 Hz can effectively remove a large portion of physiological noises including heartbeat and Mayer waves without eliminating the fNIRS signal elicited by a task of 10 s period. The types of band-pass filtering include Butterworth filters (Luu and Chau, 2009; Naseer and Hong, 2013; Naseer et al., 2014), elliptic filters (Hu et al., 2012), and Chebyshev filters (Sitaram et al., 2007; Power et al., 2012b). However, no absolute advantage of a particular filtering method over others has been reported yet.

### Advanced filtering methods

Band-pass filtering cannot be used to filter physiological noises whose frequencies overlap with the band of the hemodynamic response signal, for example, due to respiration. Therefore, other methods, such as adaptive filtering (Zhang et al., 2007; Hu et al., 2010; Aqil et al., 2012a,b; Kamran and Hong, 2013, 2014), PCA (Zhang et al., 2005), and ICA (Kohno et al., 2007; Santosa et al., 2013), have also been used to remove physiological noise. To account for physiological noises, additional noise-related elements can be added into the regression model. In addition to modeling the canonical functional response, a series with adaptive amplitudes and phase components in order to model specific physiological noise contribution from heartbeat, respiration, and blood pressure can be included. The auto-regressive moving average with exogenous signals (ARMAX) model-based approach incorporating physiological signals as exogenous signals can be used to predict the brain state during a particular cognitive task. The fNIRS signal at each channel can be regarded as an output from a linear combination of various components. The components include the dynamical characteristics of the HbO and HbR changes in a specific brain region (the influence from the current/previous stimuli), the physiological signals, the baseline fluctuation, and other noises.

### ICA and PCA

ICA can separate physiological noises from the mixed signals allowing the restoration of the original hemodynamic signals. The independent components (ICs) associated with the physiological signals can be identified by their spectral densities. Isolating the main IC associated with the original hemodynamic response results in a physiological-noise-free signal. Hu et al. (2011) and Santosa et al. (2013) used ICA to separate physiological noise from the original signals. Then, the original hemodynamic response was reconstructed using all the ICs (with weights derived from their *t*-values) as well as the primary IC. They applied the proposed method to a mental arithmetic task and compared the results with those of the conventional low-pass filtering method, revealing that the ICA method outperformed the low-pass filtering method. Funane et al. (2014) used ICA to evaluate signal contributions from the shallow and deep tissue layers using multi-distance optodes. They assumed that the optical path length in the shallow layer did not change, but it increased linearly with the increase of emitter-detector distance. The reconstructions of the deep and shallow layer signals were performed by summing all the ICs that had been weighted by the deep and shallow contribution ratio in accordance with the emitter-detector distance.

PCA can be used to remove physiological noises (similarly to the case of motion-artifact removal), because systematic fluctuations are covariant among fNIRS measurements from different channels. Reducing such covariance, accordingly, filters systematic physiological noises from the signals. However, the performance of PCA is greatly dependent on the number of channels and the number of eigenvectors to be removed (Cooper et al., 2012) and, therefore, PCA is not suggested for physiological noise removal when the number of channels is small. Furthermore, a real-time application of ICA for physiological noise removal is still under investigation (a moving window approach for computing ICs can be explored). Henceforth, due to the non-realtimeness of the ICA approach, band-pass filtering techniques are still dominant (Mihara et al., 2012, 2013; Kober et al., 2014).

The fNIRS signals are also affected by the skin blood flow and other contributions from the superficial tissues (Kohno et al., 2007; Takahashi et al., 2011; Kirilina et al., 2012, 2013; Sato et al., 2013). It has been shown that the removal of these artifacts from cerebral signals is possible by employing several different methods: the use of additional short-distance detector(s) (Saager and Berger, 2005; Luu and Chau, 2009; Saager et al., 2011), adaptive filtering (Zhang et al., 2009), statistical parametric mapping (SPM) in which the artifacts are included as regressors into the model (Tachtsidis et al., 2010), and ICA (Kohno et al., 2007; Funane et al., 2014). Kohno et al. (2007) revealed that the spatial distribution of one of the ICs was directly related to the skin blood flow, which was again verified by a laser Doppler tissue blood flow meter. Funane et al. (2014), on the other hand, used ICA to separate the absorption changes in deep and shallow tissues (due to the scalp and the skin) using multiple emitter-detector distances. Zhang et al. (2007, 2009) used an adaptive filter to estimate the global interference in the signals measured from short emitter-detector separations. This global interference was then removed from the target signals measured from long emitter detector separations.

## Feature extraction/selection

After data preprocessing, the different brain activities are classified on the basis of certain features. In fNIRS-BCI, although some features are extracted directly from detected light-intensity signals (Naito et al., 2007; Luu and Chau, 2009; Power et al., 2010, 2011), most are extracted from hemodynamic signals. The reason for this is that HbO, HbR, total hemoglobin (HbT), and cerebral oxygen exchange (COE = HbO - HbR) provide more options for selection of appropriate features. Selection of an optimal feature set for classification is essential for good classification. It is necessary to select such features that have similarities with a certain class and differences from other classes. Different combinations of such features provide the necessary discriminatory information for classification.

### Heuristic methods

After noise removal, the shape of the hemodynamic signal is usually clear. By observing the hemodynamic signals arising from different activities, one can determine the differences in the signals: peak amplitude, mean value, variance, slope, skewness, kurtosis, etc. These can then be used as features for classification of different signals. The most commonly used features for discrimination of different activities for fNIRS-BCI are signal mean (Coyle et al., 2004, 2007; Sitaram et al., 2007; Luu and Chau, 2009; Power et al., 2010; Holper and Wolf, 2011; Fazli et al., 2012; Moghimi et al., 2012; Faress and Chau, 2013; Naseer and Hong, 2013; Naseer et al., 2014; Hong et al., 2015), signal slope (Power et al., 2011, 2012a,b; Hai et al., 2013; Naseer and Hong, 2013; Power and Chau, 2013; Schudlo and Chau, 2014; Hong et al., 2015), signal variance (Tai and Chau, 2009; Holper and Wolf, 2011), amplitude (Naito et al., 2007; Cui et al., 2010b; Bauernfeind et al., 2011; Stangl et al., 2013), skewness (Tai and Chau, 2009; Holper and Wolf, 2011), kurtosis (Tai and Chau, 2009; Holper and Wolf, 2011), and zero crossing (Tai and Chau, 2009).

### Filter coefficients

Some fNIRS-BCI studies have proposed the use of filter coefficients (as classification features) obtained by Kalman filtering (Abdelnour and Huppert, 2009), recursive least square estimation (Aqil et al., 2012a), and wavelet transform (Khoa and Nakagawa, 2008; Abibullaev et al., 2011; Abibullaev and An, 2012). They assumed that different brain activities will produce different filter coefficients, in which different signals can be classified. This method has been shown to work well, even though no significant classification-accuracy improvement over the heuristic methods has been demonstrated.

### Genetic algorithms

Genetic algorithms are an optimization technique that is used to select the most efficient features from a set. Power et al. (2012a) used a genetic algorithm to select features by employing LDA as a fitness function. For more details on genetic algorithms, please see Pernkopf and O'Leary (2001) and Nicolas-Alonso and Gomez-Gil (2012).

Although feature selection is also dependent on individual activities, the mean values and slope values of HbO, HbR, or HbT frequently have been used in fNIRS-BCI. Almost half of fNIRS-BCI studies have used either the mean value or the slope value of the signal as one of the features for classification. It has been shown that HbO performs more robustly than HbR and HbT for assessing task-related cortical activation (Mihara et al., 2012; Naseer and Hong, 2013; Naseer et al., 2014). Plichta et al. (2006) showed that the retest reliability and stability over time of HbO signals are higher than those of HbR signals. From the above reasons, feature extraction using HbO signals is more suitable for classification in fNIRS-BCI.

## Classification techniques

Classification techniques are used to identify the different brain signals that are generated by the user. These identified signals are then translated into control commands for application interface purposes. In most existing fNIRS-BCIs, such identification is performed by using classification techniques to discriminate various brain signals based on appropriate features. Classification algorithms, as calibrated by the users through supervised learning during the training phase, are able to detect brain-signal patterns during the testing stage. Some of the commonly used classification methods in fNIRS-BCI are LDA, SVM, HMM, and artificial neural networks (ANN).

### LDA

**Figure 3:**
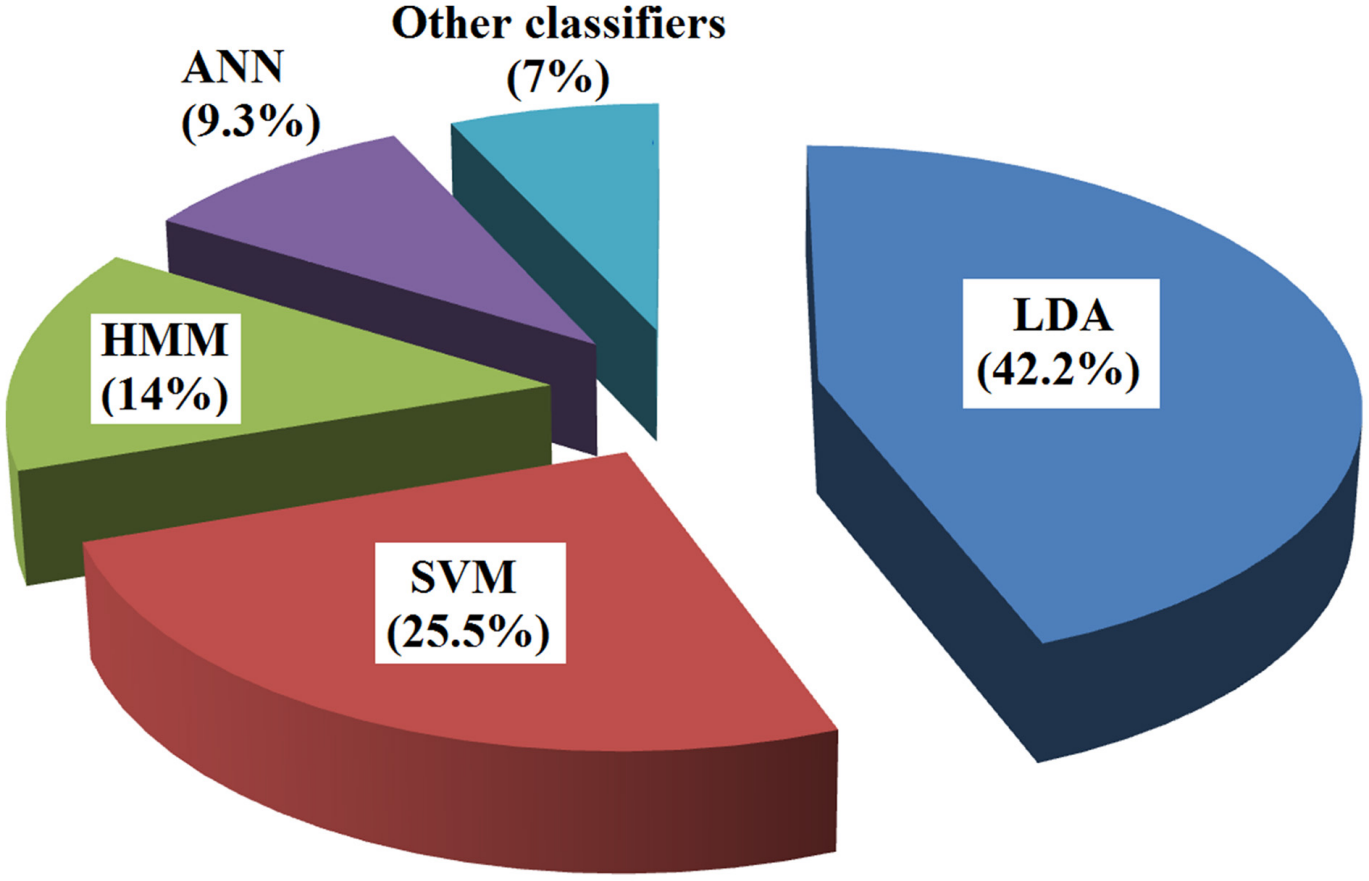
**Types of classifiers in fNIRS BCI (from 2004 to 2014)**.

**Figure 4:**
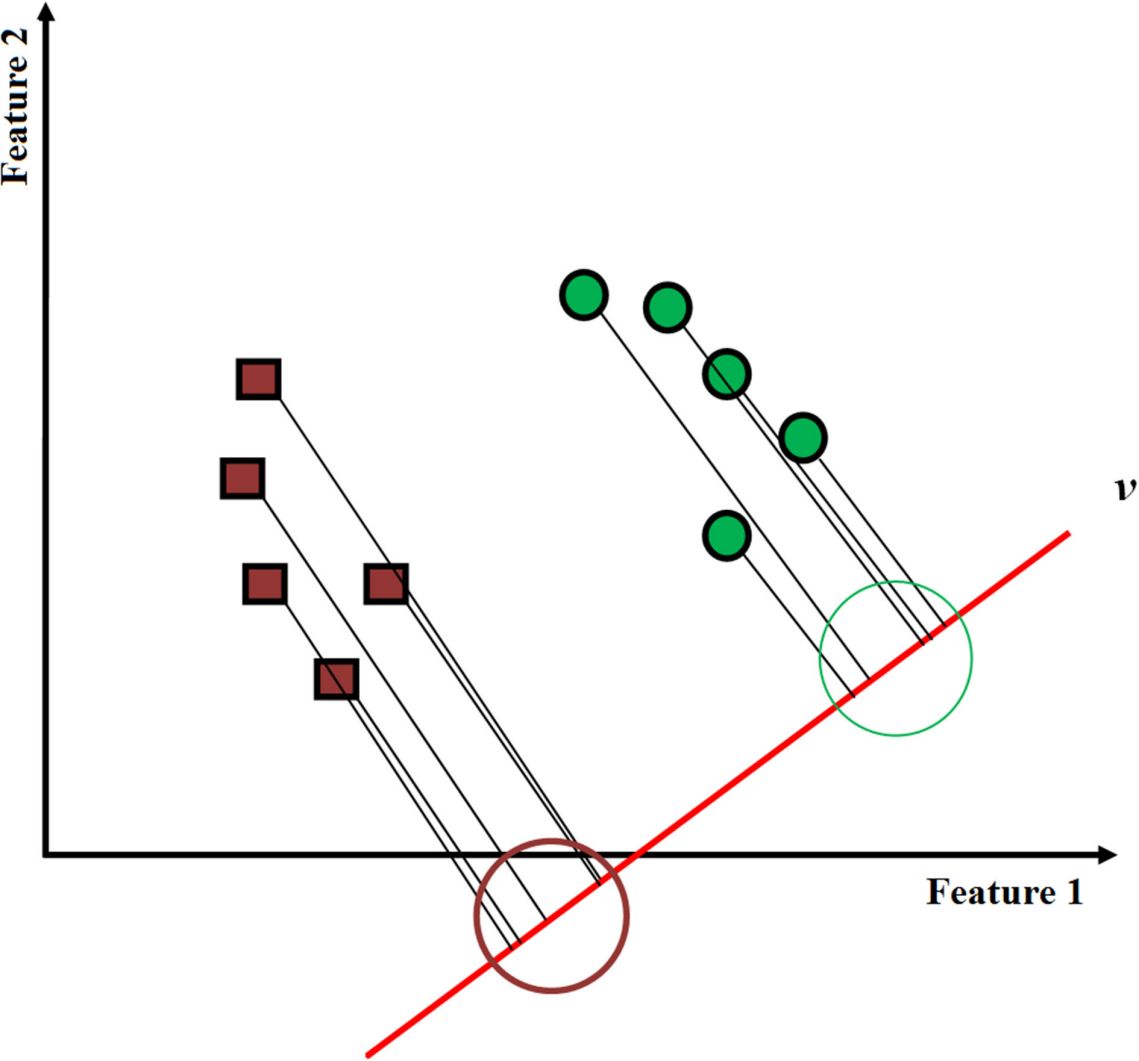
**LDA classification depicting the best separating hyperplane**.

LDA is the most commonly used classification in fNIRS-BCI studies (see Figure 3). It utilizes discriminant hyperplane(s) to separate data representing two or more classes. Because of its simplicity and low computational requirements, it is highly suitable for online BCI systems. Not surprisingly, it has been used in a number of fNIRS-BCI studies. In LDA, the separating hyperplane is found by seeking such data projection by maximizing the distance between the two classes' means and minimizing the interclass variances. LDA assumes a normal data distribution along with an equal covariance matrix for both classes (Lotte et al., 2007). An LDA algorithm tries to find a vector *v* in the feature space such that two projected classes 1 and 2 in the *v*-direction can be well separated from each other while maintaining a small variance for each (see Figure 4). This can be accomplished by maximizing the Fisher's criterion given by:

(1) $$J(v)=\frac{v^{\text{T}}S_{\text{b}}v}{v^{\text{T}}S_{\text{w}}v}$$

where *S*_*b*_ and *S*_*w*_ are the between-class and within-class scatter matrices defined as:

(2) $$S_{\text{b}}\;=\left(m_{1}-m_{2}\right){\left(m_{1}-m_{2}\right)}^{\text{T}},$$

(3) $$S_{\text{w}}\;=\sum_{x_{n}\in \text{ C1}}\text{​}\left(x_{n}\text{​}-\text{​}m_{1}\right){\left(x_{n}\text{​}-\text{​}m_{2}\right)}^{\text{T}}\text{​}+\text{​}\sum_{x_{n}\in \text{ C2}}\text{​}\text{​}\left(x_{n}\text{​}-\text{​}m_{1}\right){\left(x_{n}\text{​}-\text{​}m_{2}\right)}^{\text{T}}$$

where *m*_1_ and *m*_2_ represent the group means of classes C1 and C2, respectively, and, *x*_*n*_ denotes samples. It can be seen that a vector *v* that satisfies (1) can be reformulated as a generalized eigenvalue problem as:

(4) $$S_{\text{w}}^{-1}S_{\text{b}}v=\lambda v.$$

The optimal *v* is then the eigenvector corresponding to the largest eigenvalue of *S*^−1^_w_*S*_b_ or is directly obtained as:

(5) $$v=S_{\text{w}}^{-1}(m_{1}-m_{2})$$

provided that *S*_w_ is non-singular.

Many fNIRS studies have successfully demonstrated the use of LDA for BCI (Luu and Chau, 2009; Bauernfeind et al., 2011; Holper and Wolf, 2011; Power et al., 2011, 2012a,b; Abibullaev and An, 2012; Fazli et al., 2012; Moghimi et al., 2012; Faress and Chau, 2013; Naseer and Hong, 2013; Power and Chau, 2013; Stangl et al., 2013; Kaiser et al., 2014; Naseer et al., 2014; Schudlo and Chau, 2014; Hong et al., 2015).

### SVM

**Figure 5:**
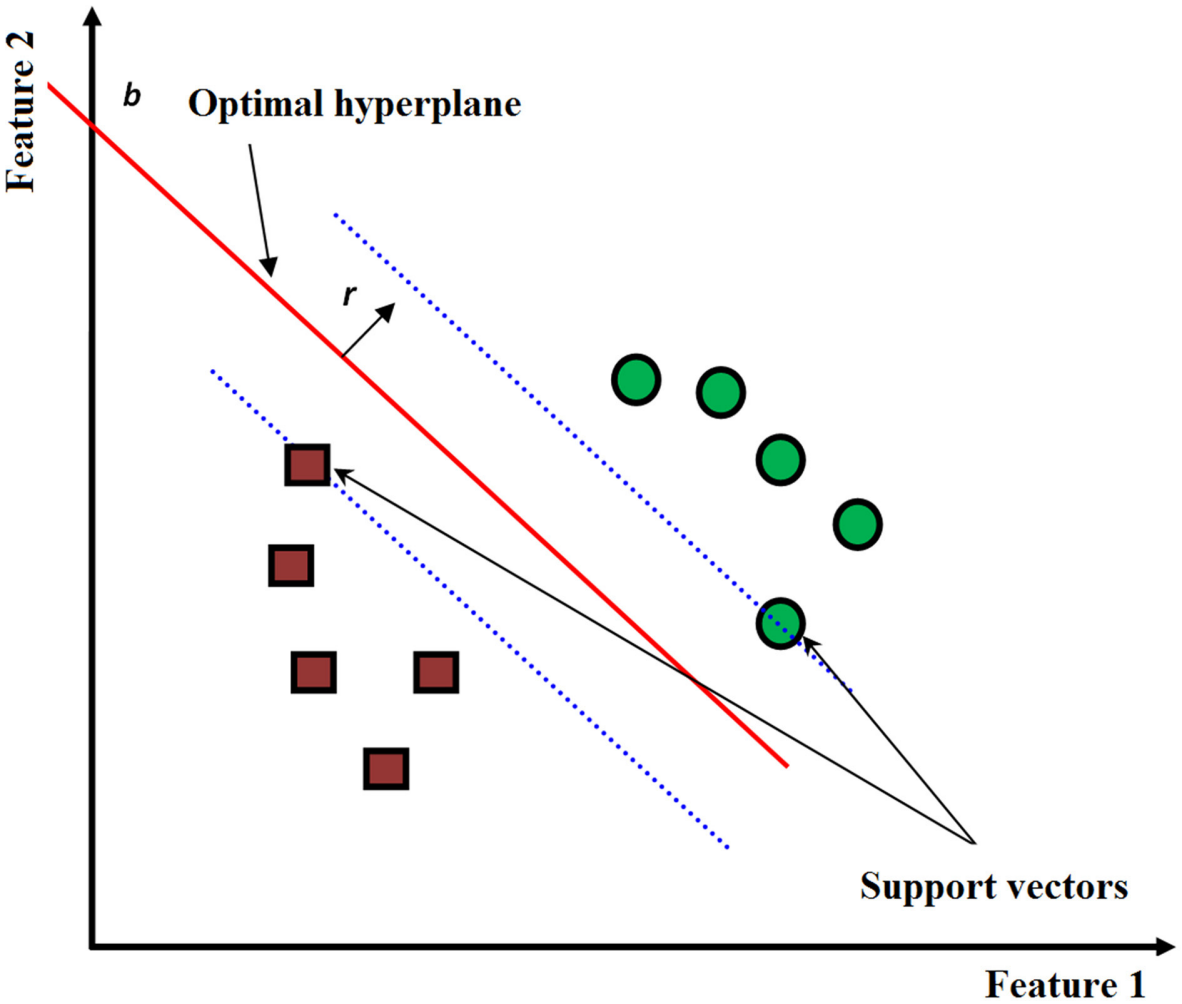
**SVM classification illustrating the optimal hyperplane that maximizes the distance from the nearest support vectors**.

The SVM classifier tries to maximize the distance between the separating hyperplane and the nearest training point(s) (the so-called support vectors) (see Figure 5). The separating hyperplane in the 2D feature space is given by the equation:

(6) f(x)=r.x+b,

where *r*, *x*∈*R*^2^ and *b*∈*R*^1^ (see Figure 5). The optimal solution r^*^ that maximizes the distance between the hyperplane and the nearest training point(s) can be obtained by minimizing the cost function.

(7) $$J\left(r,\xi \right)=\frac{1}{2}{\left\|r\right\|}^{2}+C.\sum_{n=1}^{z}\xi_{n},$$

while satisfying the constraints:

(8) $$\begin{array}{l} \;\left(x_{n}.r+b\right)\geq 1-\xi_{n}\text{ for }y_{n}=+1,\\ \left(x_{n}.r+b\right)\geq -1+\xi_{n}\text{ for }y_{n}=-1,\\ \;\xi_{n}\geq 0\;\forall \;n, \end{array}$$

where ║*r*║^2^ = *r*^*T*^*r*, *C* is the positive regularization parameter chosen by the user (a large value of *C* corresponds to a higher penalty for classification errors), ξ_n_ is the measure of training error, *z* is the number of misclassified samples, and *y*_*n*_ is the class label (+1 or −1 in the case of binary classification) for the *n*-th sample.

Since SVM maximizes the distance from the nearest training point(s), it is known to enhance the generalization capabilities. Also, the regularization parameter *C* allows for accommodating the outliers and therefore reduces errors on the training sets (Burges, 1998). Although SVM is a linear classifier because it uses one or more hyperplanes, it is possible to make SVM with non-linear decision boundaries. This can be done by using kernel functions such as the Gaussian or radial basis functions (known commonly as RBF). Non-linear SVM provides a more flexible decision boundary that can result in an increased classification accuracy. Using the kernel functions might, however, be computationally more demanding.

SVM has been shown to work well in a number of fNIRS-BCI studies (Sitaram et al., 2007; Tai and Chau, 2009; Cui et al., 2010b; Tanaka and Katura, 2011; Abibullaev and An, 2012; Hu et al., 2012; Misawa et al., 2012; Hai et al., 2013; Naseer et al., 2014).

### ANN

ANNs are non-linear classifiers that have been used in a few fNIRS-BCI studies (Abibullaev et al., 2011; Chan et al., 2012; Hai et al., 2013). ANNs were inspired by the fact that the human and animal brains are able to react adaptively to changes in internal and external environments. An appropriate model of the nervous system can produce a similar process in an artificial system. ANNs therefore try to mimic brain activity to solve problems. ANNs are widely used in pattern recognition problems, owing to their post-training capability to recognize sets of training-data-related patterns. ANNs consist of assemblies of several artificial neurons that allow for the drawing of non-linear decision boundaries. They can be used in several different architectures including multilayer perception, Gaussian classifier, learning vector quantization, RBF neural networks, and others. For more details on these architectures, please see (Anthony and Bartlett, 2009).

### HMM

HMM is a non-linear probabilistic classifier that provides the probability of observing a given set of features that are suitable primarily for classification of time series (Rabiner, 1989). Some fNIRS studies, for example, have successfully demonstrated the feasibility of using HMM for BCI (Sitaram et al., 2007; Power et al., 2010; Falk et al., 2011; Chan et al., 2012; Zimmermann et al., 2013).

Two other classifiers that have been used in fNIRS-BCI are partial least squares discriminant analysis (PLSDA) (Seo et al., 2012) and quadratic discriminant analysis (QDA) (Naito et al., 2007). Although some non-linear classifiers have been shown to increase classification accuracies over those of linear classifiers, the high-speed execution of the linear classifiers has made them the preferred ones for fNIRS-BCI. Almost 45% of fNIRS-BCI studies have utilized LDA for classification (see Figure 3), due specifically to its fine balance between the classification accuracy and the execution speed.

## fNIRS-BCI applications

In recent years, significant progress has been made in fNIRS-BCI research; however, the applications have been designed mostly for training and demonstration purposes only. fNIRS-BCI has two main drawbacks that have limited its use in real-world applications: a slow information transfer rate, and high error rates. Another problem is the fact that most fNIRS-BCIs are tested in controlled laboratory environments where the user can comfortably concentrate well on mental tasks; whereas in real situations, performance of concentration-dependent mental tasks (e.g., motor imagery, mental arithmetic, etc.) is much more challenging.

### Neuro-rehabilitation

BCI systems can be used to restore some of the lost motor and/or cognitive functions in individuals with stroke and spinal cord injury. The underlying idea of doing so is the ability of BCI feedback to induce self-regulation of brain activity. EEG, due to its high temporal resolution, has been used in a large number of previous neurofeedback studies (please see Gruzelier, 2013, for a review of EEG-based neurofeedback studies). However, since EEG has the limitations of imprecise localization and inaccessibility of subcortical areas, the hemodynamic activity measured by fMRI has been used in neurofeedback studies to overcome these problems. A comprehensive review of fMRI-based BCI and neurofeedback studies is provided by LaConte (2011) and Weiskopf (2012).

fNIRS is very attractive, in comparison with fMRI, in accessing subcortical brain signals. It is low cost, easy to use, and most of all it is portable. It can be used even in an ambulance. It also has a better temporal resolution than most of fMRI scanners (Huppert et al., 2006). Moreover, fNIRS is less sensitive to motion artifacts because it can be attached (or worn) to the brain or on the body. Given the above points, the potential of use of fNIRS in neurofeedback studies is very high. Mihara et al. (2012) demonstrated the possibility of using fNIRS-based neurofeedback to allow the users to willfully regulate their hemodynamic responses. They also showed that fNIRS-based neurofeedback enhances the hemodynamic correlates corresponding to motor imagery. Further, the same group have also reported similar results for stoke patients (Mihara et al., 2013). Recently, Kober et al. (2014) revealed that fNIRS-based neurofeedback can be used for a long-term training as well, and such repetitive neurofeedback can induce specific and focused brain activation: In contrast, sham feedback has led to diffuse brain activation patterns over broader brain areas. One important disadvantage of using hemodynamics (either fMRI or fNIRS) for neurofeedback is the inherent delay in its response, which makes the generation of commands slow compared to EEG. However, in the case of fNIRS, this kind of disadvantage can be solved if the initial dip (i.e., the phenomenon that HbO decreases and HbR increases with neural firing) can be measured (instead of hemodynamics).

### Communication

The primary application of BCI is to serve as a means of communication for people with motor disorders such as ALS, spinal cord injury and/or who are suffering from a persistent LIS. Naito et al. (2007) and Naseer et al. (2014) developed an fNIRS-BCI system for binary communication based on activations from the prefrontal area. The subjects were required to perform a specific task such as mental arithmetic or music imagery to increase the cognitive load and, thereby, respond “yes” or to remain relax and, thus, respond “no” to the given question. The average accuracies obtained by Naseer et al. (2014) with online classification were approximately 82%. Sitaram et al. (2007) proposed an fNIRS-BCI-based online word speller. Their system involves using right-hand and left-hand motor imagery to move a cursor on a two-dimensional to select letters.

### Motor restoration/rehabilitation

Another important application of fNIRS-BCI is the restoration of movement capability for people with motor disabilities. The control commands generated by a BCI system can be used to control a prosthetic limb or a wheelchair. It is desirable to have a portable system for these applications so that the user can move freely. Also these applications, for safety purposes, cannot afford high error rates, and must be fast enough to provide real-time control. Several fNIRS-BCI studies have tried to improve classification accuracies and information transfer rates (Shin and Jeong, 2014). Using neurofeedback, induction of neuroplasticity of selected brain areas which has the potential to improve cognitive performance, also can be accomplished.

### Other applications

Other applications of fNIRS-BCI include environment control and entertainment. Environment control applications (for instance, remote control, lights and temperature control) are very useful for motor-disabled people. Recently, BCI has also been used for healthy individuals' entertainment purposes, although this is not a main priority of BCI research. The feasibility of brain-controlled video games has been demonstrated using EEG-BCI; however, no such fNIRS-based application has been introduced to date. For training purposes though, such games might be useful.

Table 1 provides a summary of most studies published from 2004 to 2014 that demonstrated important roles in brain-signal-acquisition, signal pre-processing, feature-selection, and classification stages for fNIRS-BCI.

**Table 1:** **Important fNIRS-BCI studies from 2004 to 2014**.

**References**	**Brain area**	**Task**	**Filter used for noise removal**	**Features**	**Classifier**	**Classification accuracy (%)**
Coyle et al., 2004	Motor cortex	Motor imagery	Low-pass	Mean values of ΔHbO	Threshold-based	75
Sitaram et al., 2007	Motor cortex	Motor imagery	Low-pass	Mean of ΔHbO and ΔHbR for all channels	SVM and HMM	73 (SVM)
						89 (HMM)
Naito et al., 2007	Prefrontal cortex	Mental arithmetic, music imagery and landscape imagery	Low-pass	Amplitude of light intensity	QDA	80
Coyle et al., 2007	Motor cortex	Motor imagery	Low-pass	Mean values of ΔHbO	Threshold-based	80
Luu and Chau, 2009	Prefrontal cortex	Neural correlates of subjective preference	Low-pass	Mean amplitude of light-intensity signals	LDA	80
Tai and Chau, 2009	Prefrontal cortex	Emotion rehearsal associated with images	Least-mean square adaptive filter	Mean, variance, zero crossing, root mean square, skewness and kurtosis of ΔHbO and ΔHbR	LDA and SVM	96.6 (LDA)
						94.6 (SVM)
Power et al., 2010	Prefrontal cortex	Mental arithmetic/Music imagery	Low-pass/Wavelet filter	Mean values of light intensity	HMM	77.2
Cui et al., 2010b	Motor cortex	Finger tapping	Exponential moving average filter	Amplitude, history, history gradient and 2nd order gradient of ΔHbO and ΔHbR, spatial patterns	SVM	>80 (spatial patterns)
Abibullaev et al., 2011	Prefrontal cortex	Object rotation, verbal fluency and mental arithmetic	Wavelet filter	Mean, power, standard deviation etc. of the filter coefficients from wavelet transform	ANN	> 94
Falk et al., 2011^*^	Prefrontal cortex	Music imagery	Low-pass	ΔHbO and ΔHbR values after wavelet transform	HMM	83
Holper and Wolf, 2011	Motor cortex	Motor imagery	Low-pass	Mean, variance, skewness and kurtosis of ΔHbO	LDA	81
Tanaka and Katura, 2011	Prefrontal and Visual cortex	Change-detection task	Moving average/ Band-select	ΔHbO and ΔHbR values from single, two and three channels	SVM	77
Bauernfeind et al., 2011	Prefrontal cortex	Mental arithmetic	High-pass	Antagonistic ΔHbO signals	LDA	79.7
Power et al., 2011	Prefrontal cortex	Mental arithmetic/Mental singing	Low-pass	Slope of light-intensity signals	LDA	71.2 (mental arithmetic)
						62.7 (mental singing)
Chan et al., 2012	Prefrontal cortex	Mental singing	Low-pass	ΔHbO and ΔHbR signals from selected channels	ANN and HMM	63 (ANN)
						55.7 (HMM)
Fazli et al., 2012^**^	Motor cortex	Motor execution and motor imagery	Low-pass	Mean values of ΔHbO and ΔHbR and band power of Laplacian filtered EEG data	LDA	92.6 (EEG+HbR for motor execution)
						83.1 (EEG+HbR for motor imagery)
Seo et al., 2012	Motor cortex	Finger tapping	Moving average/band-pass	Raw ΔHbO values at three time points	PLSDA and HMM	90.2 (PLSDA)
						85.7 (HMM)
Hu et al., 2012	Prefrontal cortex	Deception	Band-pass	Absolute values of ΔHbO and ΔHbR	SVM	83.4
Power et al., 2012b	Prefrontal cortex	Mental arithmetic	Low-pass	Signal slope	LDA	72.6
Moghimi et al., 2012	Prefrontal cortex	Emotionally rated music listening	Low-pass	Mean and difference between signal and noise of ΔHbO and ΔHbR	LDA	71.9
Abibullaev and An, 2012	Prefrontal cortex	Object rotation, letter padding and multiplication	Wavelet filter	Filter coefficients from wavelet transform	ANN, LDA and SVM	>75 (ANN)
						>85 (LDA)
						>90 (SVM)
Liu et al., 2013	Prefrontal cortex	Neural correlation of visual stimulus	Band-pass	Mean values of ΔHbO and ΔHbR and EEG amplitudes	Step-wise LDA	>90
Power and Chau, 2013	Prefrontal cortex	Mental arithmetic	Low-pass	Signal slope of ΔHbO and ΔHbR	LDA	71.1
Stangl et al., 2013	Motor cortex, prefrontal cortex	Motor imagery, mental arithmetic	Moving average	Amplitude of ΔHbO	LDA	65
Zimmermann et al., 2013^*^	Motor cortex	Motor execution	Band-pass	Combination of ΔHbO and ΔHbR and biosignals belonging to same location and same time	HMM	88.5
Naseer and Hong, 2013	Motor cortex	Motor imagery	Low-pass	Mean and slope values of ΔHbO and ΔHbR in several temporal windows	LDA	77.5 (mean value)
						87.2 (signal slope)
Faress and Chau, 2013^***^	Prefrontal cortex	Verbal fluency	Low-pass	Slope of HbO, HbR and HbT	LDA	86
Hai et al., 2013	Motor cortex	Hand tapping	Savitzky-Golay	Signal values after polynomial regression	SVM and ANN	79.1 (SVM)
						83.3 (ANN)
Schudlo and Chau, 2014	Prefrontal cortex	Mental arithmetic	Low-pass	Slope of ΔHbO, ΔHbR and ΔHbT in 3 different time windows	LDA	77.4
Naseer et al., 2014	Prefrontal cortex	Mental arithmetic	Low-pass	Mean values of ΔHbO and ΔHbR	LDA and SVM	74.2 (LDA)
						82.1 (SVM)
Khan et al., 2014^**^	Motor cortex, prefrontal cortex	Motor execution, mental counting and mental arithmetic	Band-pass	Mean values of ΔHbO and ΔHbR	LDA	>80
Shin and Jeong, 2014	Motor cortex	Motor execution	Band-pass and Savitzky-Golay	Mean, amplitude, slope, delay, variance and median	Naïve Bayes classifier	95.5 (binary)
						92.4 (ternary)
						91.5 (quaternary)
Hwang et al., 2014	Motor cortex, Prefrontal cortex	Motor Imagery, mental singing, mental arithmetic, mental rotation and mental character writing	Band-pass	Mean values of HbO, HbR and HbT	LDA	>70 (mental arithmetic and mental rotation)
Hong et al., 2015	Motor cortex, prefrontal cortex	Motor imagery, mental arithmetic	Band-pass	Mean and slope of HbO	Multi-class LDA	> 75

* For classification purpose, they also used some additional features from ECG, respiration, blood pressure, skin conductance response, etc.

** They used combined fNIRS and EEG modalities for brain signal acquisition.

*** This study used additional transcranial Doppler ultrasonography signals together with fNIRS signals.

## Future prospects of fNIRS-BCI

Given the advantages (non-invasive, cheap, portable, and silent), the use of fNIRS for BCI purposes is more suitable than fMRI. Furthermore, its use is easier than EEG that uses wet electrodes. A limitation of using fNIRS for BCI is that the information transfer rate is limited by the inherent delay in the hemodynamic response. However, the detections of the fast optical response (Gratton et al., 2006; Hu et al., 2011) and the initial dip (Akin et al., 2006; Yoshino and Kato, 2012) have been demonstrated, which can offer faster information transfer rate and better control. Since the speed of EEG can be utilized, the authors believe that the future of non-invasive, portable and wearable BCIs lies in the use of hybrid EEG-fNIRS systems, as it has shown to work superior to EEG-BCIs and fNIRS-BCIs alone (Fazli et al., 2012; Kaiser et al., 2014; Khan et al., 2014; Koo et al., 2014). The reason for using a hybrid or combined fNIRS-EEG system is that it either improves the classification accuracy or increases the number of control commands for BCI. This can be done by extracting some relevant features from fNIRS and combining them with EEG system. Fazli et al. (2012) demonstrated significantly enhanced performance, in terms of classification accuracy, by combined feature sets from both fNIRS and EEG. Tomita et al. (2014) showed that an optimal time slot for command generation can be estimated using indications from fNIRS signals in hybrid fNIRS-EEG. Khan et al. (2014) demonstrated an efficient control strategy for active BCI by placing fNIRS and EEG at different brain locations. Koo et al. (2014) have also shown that the self-paced motor imagery can be detected more efficiently using a hybrid fNIRS-EEG system. Since the information contents of EEG and fNIRS are very distinctive, the hybrid fNIRS-EEG system has a strong potential for future neurorehabilitation and neurofeedback applications.

## Conclusions

In this paper, we have reviewed the state-of-the-art of fNIRS-based BCI systems, discussing all the procedures appearing in the standard BCI. Several different brain activities have been used for fNIRS-BCI, including, most commonly, those from the motor and prefrontal cortices. Motor cortex activities such as motor execution and motor imagery have been shown to work well and, indeed, are useful from the neurorehabilitation perspective. Prefrontal activities, on the other hand, offer the advantages of being free from artifacts due to hair. Both, despite of their drawbacks, have been shown to work well for fNIRS-BCI purposes. Use of other brain-imaging modalities, such as EEG in combination with fNIRS in a hybrid fashion, has been shown to effectively improve BCI performance. Such hybrid systems can acquire brain signals from the same as well as different brain areas, thereby increasing the number of control commands.

Different signal-processing and noise-removal methods including band-pass filtering, ICA, principle component analysis, wavelet transform and adaptive-filtering-based methods have been discussed. Because band-pass filters are simple and incur only low computational costs, they are still mostly used in fNIRS BCI.

BCI-applied classification algorithms must be both accurate and fast. Although SVM, hidden Markov models, and artificial neural networks provide good classification accuracies, the linear discriminant analysis (in its simple structure) has a low computational cost and also provides a good performance in classification accuracy.

Considering all these points, it is concluded that there is much room for future fNIRS-BCI research, particularly in its applications. Although fNIRS-BCI applications for communication and control have been demonstrated in a number of studies, no commercial fNIRS-BCI application currently is available. All of the relevant research trends predict that interest in fNIRS-BCI will continue to grow. In the near future, several breakthroughs via bundled-type fNIRS probes, hybrid EEG-fNIRS, and detection of the initial dip are expected.

### Conflict of interest statement

The authors declare that the research was conducted in the absence of any commercial or financial relationships that could be construed as a potential conflict of interest.
