# Corrigendum “fNIRS-based brain-computer interfaces: a review”

**DOI:** 10.3389/fnhum.2015.00172

**Published:** 2015-03-26

**Authors:** Noman Naseer, Keum-Shik Hong

**Affiliations:** ^1^Department of Cogno-Mechatronics Engineering, Pusan National UniversityBusan, South Korea; ^2^School of Mechanical Engineering, Pusan National UniversityBusan, South Korea

**Keywords:** brain-computer interface (BCI), functional near-infrared spectroscopy (fNIRS), feature extraction, feature classification, physiological noise, brain-machine interfaces

In the References section of this article, Burges (1998) and Liu et al. (2013) were cited as follows.

Burges, C. J. C. (1998). A tutorial on support vector machines for pattern recognition. *Knowl. Discov. Data Min.* 2, 121–167. doi: 10.1023/A:1009715923555

Liu, Y., Ayaz, H., Curtin, A., and Onarall, B. (2013). “Towards a hybrid P300-based BCI using simultaneous fNIR and EEG,” in *Foundations of Augmented Cognition*, eds D. Schmorrow and C. Fidopiastis (Heidelberg: Springer-Verlag), 335–344.

But, the correct citations are the following.

Burges, C. J. C. (1998). A tutorial on support vector machines for pattern recognition. *Data Min. Knowl. Discov.* 2, 121–167. doi: 10.1023/A:1009715923555

Liu, Y., Ayaz, H., Curtin, A., Onaral, B., and Shewokis, P. A. (2013). “Towards a hybrid P300-based BCI using simultaneous fNIR and EEG,” in *Foundations of Augmented Cognition*, eds D. D. Schmorrow and C. M. Fidopiastis (Heidelberg: Springer-Verlag), 335–344.

## Conflict of interest statement

The authors declare that the research was conducted in the absence of any commercial or financial relationships that could be construed as a potential conflict of interest.
